# Vasectomy and Risk of Prostate Cancer: A Systematic Review and Meta-analysis

**DOI:** 10.1016/j.euros.2022.04.012

**Published:** 2022-05-19

**Authors:** Michael Baboudjian, Pawel Rajwa, Eric Barret, Jean-Baptiste Beauval, Laurent Brureau, Gilles Créhange, Charles Dariane, Gaëlle Fiard, Gaëlle Fromont, Mathieu Gauthé, Romain Mathieu, Raphaële Renard-Penna, Guilhem Roubaud, Alain Ruffion, Paul Sargos, Morgan Rouprêt, Guillaume Ploussard, Prostate Cancer Committee of the Association Française d'Urologie CC-AFU

**Affiliations:** a Department of Urology, APHM, North Academic Hospital, Marseille, France; b Department of Urology, Fundació Puigvert, Autonoma University of Barcelona, Barcelona, Spain; c Department of Urology, La Croix du Sud Hôpital, Quint Fonsegrives, France; d IUCT-O, Toulouse, France; e Department of Urology, Medical University of Vienna, Vienna, Austria; f Department of Urology, Medical University of Silesia, Zabrze, Poland; g Department of Urology, Institut Mutualiste Montsouris, Paris, France; h Department of Urology, CHU de Pointe-à-Pitre, University of Antilles, University of Rennes, Inserm, EHESP, Irset (Institut de Recherche en Santé, Environnement et Travail) – UMR_S 1085, Pointe-à-Pitre, France; i Department of Radiotherapy, Institut Curie, Paris, France; j Department of Urology, Hôpital Européen Georges-Pompidou, APHP, Paris – Paris University – U1151 Inserm-INEM, Necker, Paris, France; k Department of Urology, Grenoble Alpes University Hospital, Université Grenoble Alpes, CNRS, Grenoble INP, TIMC-IMAG, Grenoble, France; l Department of Pathology, CHRU Tours, Tours, France; m Department of Nuclear Medicine, Scintep – Institut Daniel Hollard, Grenoble, France; n Department of Urology, CHU Rennes, Rennes, France; o AP-HP, Radiology, Pitie-Salpetriere Hospital, Sorbonne University, Paris, France; p Department of Medical Oncology, Institut Bergonié, Bordeaux, France; q Service d'urologie Centre Hospitalier Lyon Sud, Hospices Civils de Lyon, Lyon, France; r Equipe 2, Centre d’Innovation en cancérologie de Lyon (EA 3738 CICLY), Faculté de médecine Lyon Sud, Université Lyon 1, Lyon, France; s Department of Radiotherapy, Institut Bergonié, Bordeaux, France; t GRC 5 Predictive Onco-Uro, AP-HP, Urology, Pitie-Salpetriere Hospital, Sorbonne University, Paris, France; u Department of Urology, Institut Universitaire du Cancer Toulouse Oncopole, Toulouse, France

**Keywords:** Vasectomy, Prostate cancer, Meta-analysis, Localized, Advanced

## Abstract

**Context:**

Previous reports have shown an association between vasectomy and prostate cancer (PCa). However, there exist significant discrepancies between studies and systematic reviews due to a lack of strong causal association and residual confounding factors such as prostate-specific antigen (PSA) screening.

**Objective:**

To assess the association between vasectomy and PCa, in both unadjusted and PSA screen-adjusted studies.

**Evidence acquisition:**

We performed a systematic review according to the Preferred Reporting Items for Systematic Reviews and Meta-analyses. The PubMed, Scopus, and Web of Science databases were searched in January 2022 for studies that analyzed the association between vasectomy and PCa.

**Evidence synthesis:**

A total of 37 studies including 16 931 805 patients met our inclusion criteria. A pooled analysis from all studies showed a significant association between vasectomy and any-grade PCa (odds ratio [OR] 1.23; 95% confidence interval [CI], 1.10–1.37; *p* < 0.001; I^2^ = 96%), localized PCa (OR 1.08; 95% CI, 1.06–1.11; *p* < 0.00001; I^2^ = 31%), or advanced PCa (OR 1.07; 95% CI, 1.02–1.13; *p* = 0.006; I^2^ = 0%). The association with PCa remained significant when the analyses were restricted to studies with a low risk of bias (OR 1.06; 95% CI, 1.02–1.10; *p* = 0.02; I^2^ = 48%) or cohort studies (OR 1.09; 95% CI, 1.04–1.13; *p* < 0.0001; I^2^ = 64%). Among studies adjusted for PSA screening, the association with localized PCa (OR 1.06; 95% CI, 1.03–1.09; *p* < 0.001; I^2^ = 0%) remained significant. Conversely, vasectomy was no longer associated with localized high-grade (*p* = 0.19), advanced (*p* = 0.22), and lethal (*p* = 0.42) PCa.

**Conclusions:**

Our meta-analysis found an association between vasectomy and any, mainly localized, PCa. However, the effect estimates of the association were increasingly close to null when examining studies of robust design and high quality. On exploratory analyses including studies, which adjusted for PSA screening, the association for aggressive and/or advanced PCa diminished.

**Patient summary:**

In this study, we found an association between vasectomy and the risk of developing localized prostate cancer without being able to determine whether the procedure leads to a higher prostate cancer incidence.

## Take Home Message

We found a significant association between vasectomy and any-grade, localized, and advanced prostate cancer. However, the effect estimates were increasingly close to null when examining studies of robust design and high quality. Future well-designed studies with attention to potential residual confounders, which were not taken into account in large cohort studies, are needed.

## 1 Introduction

Vasectomy is the fourth most common method of contraception with increasing interest among men over the past decade. Worldwide, approximately 6–8% of couples choose this method of contraception [1]. Vasectomy is the most effective permanent male contraceptive option with failure rates <1% [2]. The high level of effectiveness and low complication rates made vasectomy the foremost utilized nondiagnostic operation performed by urologists in highly developed countries [3].

Since the first report of a positive relationship between vasectomy and prostate cancer (PCa) [4], there has been an endless debate about possible associations with conflicting results. These discrepancies are due to the paucity of documented causal associations, and possible detection biases related to PCa screening and closer follow-up among vasectomy patients, and modest clinical significance with a relative risk very often close to 1. Recently, several large, high-quality reports demonstrated conflicting results [5], [6], [7], [8], [9]. A recent meta-analysis that included the most recent reports found that vasectomy was associated with localized and advanced PCa [10]. However, outcomes by disease stage were not adjusted by prostate-specific antigen (PSA) screening. Given the potential confounding effect of follow-up PSA screening, to best inform our patients on the oncological risks associated with vasectomy, the PCa Oncology Committee of the French Association of Urology conducted a systematic review of the literature and performed a meta-analysis, with a particular focus on whether there is an association between vasectomy and PCa, in both unadjusted and PSA screening–adjusted studies.

## 2 Evidence acquisition

### 2.1 Protocol and registration

We conducted a systematic review in line with the Preferred Reporting Items for Systematic Reviews and Meta-analyses (PRISMA) guidelines [11]. A protocol was registered in PROSPERO (registration number: CRD42022303026).

### 2.2 Search strategy

A literature search was conducted until January 2022 in PubMed/Medline, Scopus, and Web of Science databases. Studies were selected if they included men of any age (patient) who underwent vasectomy (intervention) compared with those who did not undergo vasectomy (comparator). We analyzed any subsequent diagnosis of PCa (outcome) in prospective and retrospective studies (study design). The search strategy used the combination of the following terms grouped according to the Boolean operators (AND, OR, and NOT): vasectomy, deferentectomy, vasoligation, vasoligature, prostate, prostatic, neoplasm, tumor, and cancer. Initial screening was performed independently by two investigators based on the titles and abstracts of the article to identify ineligible reports (M.B. and P.R.). Reasons for exclusion were noted. Potentially relevant reports were subjected to a full-text review, and the relevance of the reports was confirmed after the data extraction process. Disagreements were resolved by consultation with a third coauthor (G.P.).

### 2.3 Inclusion and exclusion criteria

We included prospective and retrospective studies, which analyzed over 1000 patients, that compared the risk of developing PCa in vasectomized and nonvasectomized patients. No patient had a personal history of PCa at baseline. In case of duplicate publications, either the higher-quality or the most recent publication was selected. Reviews, meta-analyses, commentaries, meeting abstracts, authors’ replies, thesis, and case reports were excluded, but the reference section was checked not to omit relevant articles. Case series lacking comparator groups were also excluded. No restriction on the publication date was applied. Only English-language articles were assessed for eligibility.

### 2.4 Data Extraction

Two authors (M.B. and P.R.) performed an independent initial screening based on the titles and abstracts, and noted the cause of exclusion of ineligible reports. Studies were considered eligible if these reported an effect estimate for an association between vasectomy and any PCa incidence (detection). We independently extracted the following variables from the included studies: first author’s name, publication year, country of research, study design, period of patient recruitment, number of patients included, PSA screening, duration of follow-up, PCa detection, tumor characteristics, and potential confounders. We extracted odds ratios (ORs) with 95% confidence intervals (CIs) for the risk of developing PCa in vasectomized versus nonvasectomized patients. The primary outcome was a diagnosis of any PCa. Secondary outcomes included the diagnosis of PCa stratified by disease stages: localized PCa, localized high-grade PCa, advanced PCa, and fatal PCa. No single consensus criterion was used to define high-grade and advanced PCa, and we used the definitions reported in each included study. All discrepancies regarding data extraction were resolved by consensus with a senior author (G.P.).

### 2.5 Quality assessment and risk of bias

We used the Newcastle-Ottawa Scale (NOS) to assess studies’ quality and the risk of bias (RoB). This scale assesses RoB in three areas: study group selection, group comparability, and exposure and outcome assessment. Studies that scored ≥7 were considered of high quality, and those with scores 4–6 were of moderate quality and scores <4 of poor quality. We considered the follow-up adequate if the median or mean follow-up was >5 yr. We assessed publication biases using funnel plots.

### 2.6 Statistical analysis

We used the inverse variance technique to calculate the pooled ORs for PCa risk and corresponding 95% CIs. We assessed heterogeneity using the Q test and quantified it using I^2^ values [12]. We used either a fixed- or a random-effect model for calculations of ORs according to the heterogeneity of the pooled studies. We assessed heterogeneity using the Cochrane’s Q test and quantified it using I^2^ values. In the case of heterogeneity (Cochrane’s Q test *p* < 0.05 and I^2^ > 50%), we used a random-effect model (DerSimonian method) and attempted to investigate and explain the heterogeneity; otherwise, the fixed-effect model (Mantel-Haenszel method) was used. Meta-analyses and graph figures were generated using the Cochrane Review Manager 5.4 (RevMan 5.4; The Cochrane Centre, Copenhagen, Denmark). Statistical significance was set at *p* < 0.05.

### 2.7 Subgroup analysis

Subgroup analyses were planned a priori. First, we evaluated the outcomes in subgroups of patients who were submitted to PSA screening versus those in patients in whom PSA screening was not performed or was very uncommon. Second, we examined studies according to publication year (1990–2000, 2001–2010, and 2011–2021). Third, we limited our analyses to studies identified as having a low RoB. Fourth, we evaluated the series according to study design (cohort vs cross sectional vs case control). Finally, we compared cohort studies with a follow-up of <10 versus >10 yr.

## 3 Evidence synthesis

### 3.1 Study selection

**Fig. 1:**
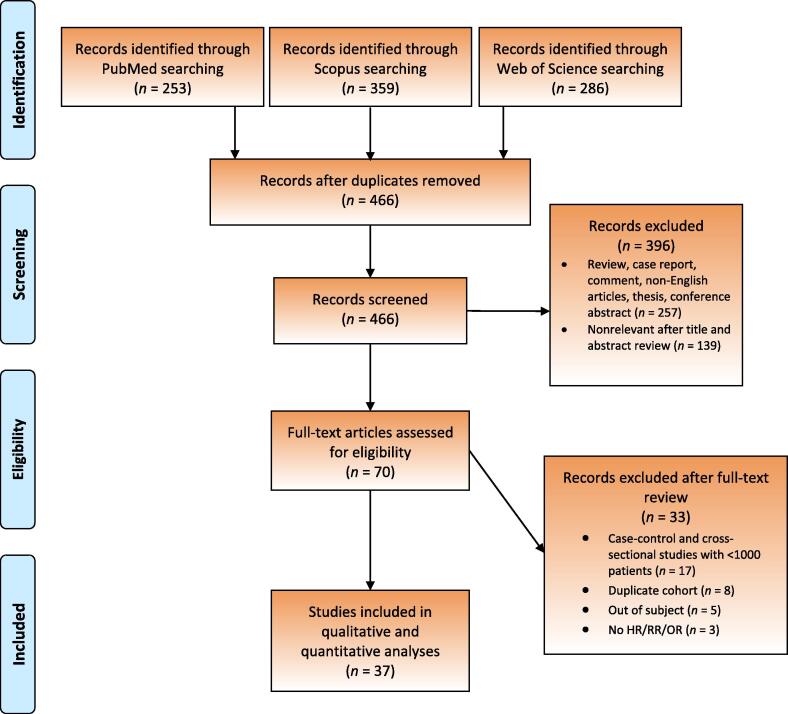
Screening process. HR = hazard ratio; OR = odds ratio; RR = relative risk.

The study selection process is outlined in the PRISMA flow diagram (Fig. 1). A total of 466 unique records were identified. Of these, 70 full-text articles were assessed for eligibility and 37 met the inclusion criteria for qualitative and quantitative analysis [4], [6], [7], [8], [9], [13], [14], [15], [16], [17], [18], [19], [20], [21], [22], [23], [24], [25], [26], [27], [28], [29], [30], [31], [32], [33], [34], [35], [36], [37], [38], [39], [40], [41], [42], [43], [44]. The reasons for exclusion are summarized in Figure 1.

### 3.2 Study characteristics

The baseline characteristics of the included studies are presented in Table 1. A total of 16 931 805 patients were included from 17 cohort studies (*n* = 4 789 366), two cross-sectional studies (*n* = 12 096 679), and 18 case-control studies (*n* = 45 760 participants). The included studies were published between 1990 and 2020, with 25 reports from North America, six from Europa, three from Asia, two from Oceania, and one from South America. Among cohort studies, follow-up varied from 4.6 to 24.8 yr and in total 80 739 patients (1.7% of all) developed PCa. The risk-adjustment approach varied considerably across studies: three were unadjusted for confounders, nine were adjusted for age, and 25 were adjusted for age and at least one other factor, including PSA screening in seven studies.

**Table 1:** Study characteristics

Study	Country of research	Study period	Study design	Number of patients included	Vasectomy procedures, *n* (%)	Follow-up, mean or median	PCa cases, *n* (%)
Smith (2017) [14]	Europe	1992–2000	Cohort study	84 753	12 712 (15)	15.4 yr	4377 (5.2)
Davenport (2019) [7]	USA	1995–2011	Cohort study	16 0571	48 657 (30.3)	18 yr	13 885 (8.6)
Eisenberg (2015) [16]	USA	2001–2009	Cohort study	873 485	112 655 (12.9)	NA	4905 (0.6)
Giovannucci (1993) [19], [20]	USA	1976–1989	Cohort study	25 340	13 034 (51.4)	11 yr	96 (0.4)
Goldacre (2005) [21]	Europe	1963–1999	Cohort study	184 253	24 773 (13.4)	12.7 yr	656 (0.4)
Hiatt (1994) [23]	USA	1979–1985	Cohort study	43 432	NA	4.6 yr	238 (0.6)
Husby (2020) [8]	Europe	1977–2014	Cohort study	2 150 162	139 550 (6.5)	24.8 yr	26 238 (1.2)
Jacobs (2016) [25]	USA	1982–2012	Cohort study	363 726 for PCa mortality	42 015 (11.6) for PCa mortality	21.4 yr for PCa mortality	9133 (13.7)
					66 542 for other outcomes	10 589 (15.9) for other outcomes	12.9 yr for other outcomes
Lynge (2002) [29]	Europe	1977–1995	Cohort study	57 931	57 931 (100)	12.7 yr	46 (0.1)
Nayan (2016) [31]	Canada	1994–2012	Cohort study	653 214	326 607 (50)	10.9 yr	3462 (0.5)
Rohrmann (2005) [34]	USA	1989–2004	Cohort study	3373	918 (27.2)	8.3 yr	78 (2.3)
Seikkula (2020) [9]	Europe	1987–2014	Cohort study	38 124	38 124 (100)	11.1 yr	413 (1.1)
Shoag (2017) [6]	USA	1993–2009	Cohort study	36 236	9933 (27.4)	13 yr	3867 (10.7)
PLCO control group							
Shoag (2017) [6]	USA	1993–2009	Cohort study	37 359	10 032 (26.9)	13 yr	4344 (11.9)
PLCO screening group							
Siddiqui (2014) [38]	USA	1986–2010	Cohort study	49 405	12 321 (24.9)	24 yr	6023 (12.2)
Tangen (2016) [41]	USA	1994–2003	Cohort study	8052	2644 (32.8)	7 yr	558 (6.9)
van Leeuwen (2011) [42]	Europe	1993–2008	Cohort study	19 950	5141 (25.8)	11.1 yr	2420 (12.1)
Alqahtani (2015) [13]	USA	2007–2011	Cross-sectional study	12 000 718	0.03% (exact number not reported)	NA	642 383
DeAntoni (1997) [17]	USA	1993–1995	Cross-sectional study	95 961	26 632 (27.8)	NA	766
Cox (2002) [15]	New Zealand	1996–1998	Case-control study	2147	549 (25.6)	NA	923
Emard (2001) [18]	Canada	1984–1993	Case-control study	6349	110 (1.7)	NA	2962
Hayes (1993) [22]	USA	1986–1989	Case-control study	2257	139 (6.2)	NA	965
Hennis (2013) [44]	Barbados	2002–2011	Case-control study	1904	1.5% of cases, 0.7% of controls (exact number not reported)	NA	963
Holt (2008) [24]	USA	2002–2005	Case-control study	1943	36% (exact number not recorded)	NA	1001
John (1995) [26]	USA/Canada	1987–1991	Case-control study	3278	336 (10.3)	NA	1642
Lesko (1999) [27]	USA	1992–1996	Case-control study	2616	414 (15.8)	NA	1216
Lightfoot (2004) [28]	Canada	1995–1999	Case-control study	2354	449 (19.1)	NA	1608
Mettlin (1990) [4]	USA	1982–1988	Case-control study	3202	154 (4.8)	NA	614
Nair-Shalliker (2017) [30]	Australia	2006–2014	Case-control study	2056	NA	NA	1181
Patel (2005) [32]	USA	1996–1998	Case-control study	1304	164 (12.6)	NA	700
Platz (1997) [33]	India	1993–1994	Case-control study	1153	100 (8.7)	NA	175
Romero (2012) [35]	Brazil	2006–2011	Case-control study	2121	259 (12.2)	NA	58
Rosenberg (1994) [36]	USA	1977–1992	Case-control study	7580	468 (6.2)	NA	553
Schwingl (2009) [37]	China/Nepal/Korea	1994–1997	Case-control study	1173	120 (10.2)	NA	294
Stanford (1999) [39]	USA	1993–1996	Case-control study	1456	562 (38.6)	NA	753
Sunny (2005) [40]	India	1998–2000	Case-control study	1170	136 (11.6)	NA	390
Weinmann (2010) [43]	USA	1974–2000	Case-control study	1697	101 (6)	NA	NA

NA = not available; PCa = prostate cancer.

### 3.3 Risk of bias

Quality and RoB assessments are summarized in Supplementary Table 1. Fourteen studies (37.8%) were assessed as having a low RoB, 20 (54.1%) as having an intermediate risk, and three (8.1%) as having a high RoB. The shape of the funnel plots was symmetric for all analyses (Supplementary Figs. 1 and 2) and only a few studies were identified over the pseudo–95% CI, indicating a low to moderate publication bias.

### 3.4 Vasectomy and any-grade cancer

**Fig. 2:**
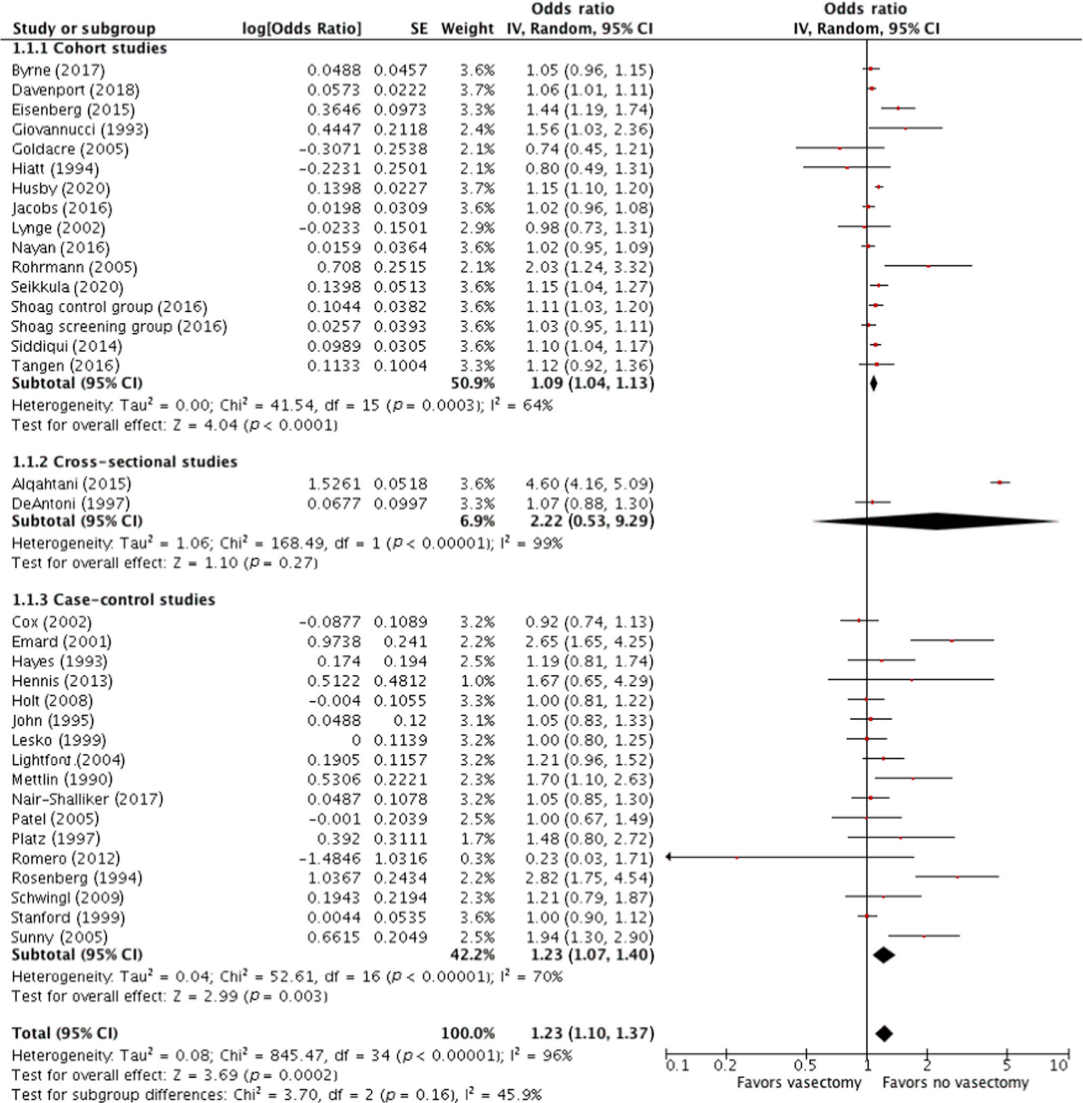
Forest plots for meta-analyses of the adjusted estimates for the association between vasectomy and prostate cancer by study design. Data were pooled separately by study design. As significant heterogeneity (I^2^ > 50%) was found, a pooled estimate was calculated with a random-effect model (DerSimonian and Laird method). CI = confidence interval; df = degrees of freedom; IV = inverse variance; SE = standard error.

The risk of PCa in vasectomized patients is presented in Figure 2. Results were first stratified by study design and then pooled. There was a significant association between vasectomy and PCa among cohort studies (OR 1.09; 95% CI, 1.04–1.13; *p* = 0.0003; I^2^ = 64%) and case-control studies (OR 1.23; 95% CI, 1.07–1.40; *p* < 0.00001; I^2^ = 96%), while the association was not significant among cross-sectional studies (OR 2.22; 95% CI, 0.53–9.29; *p* < 0.00001; I^2^ = 99%). A pooled analysis from all studies showed a significant association between vasectomy and any PCa (OR 1.23; 95% CI, 1.10–1.37; *p* = 0.0002; I^2^ = 96%). The Cochrane’s Q and I^2^ tests showed significant heterogeneity in all analyses. A sensitivity analysis was performed to assess the influence of individual studies on the overall risk of PCa. After excluding any study that did not substantially influence the direction and magnitude of the cumulative estimates, we obtained similar results.

### 3.5 Vasectomy and PCa stratified by disease stage

**Fig. 3:**
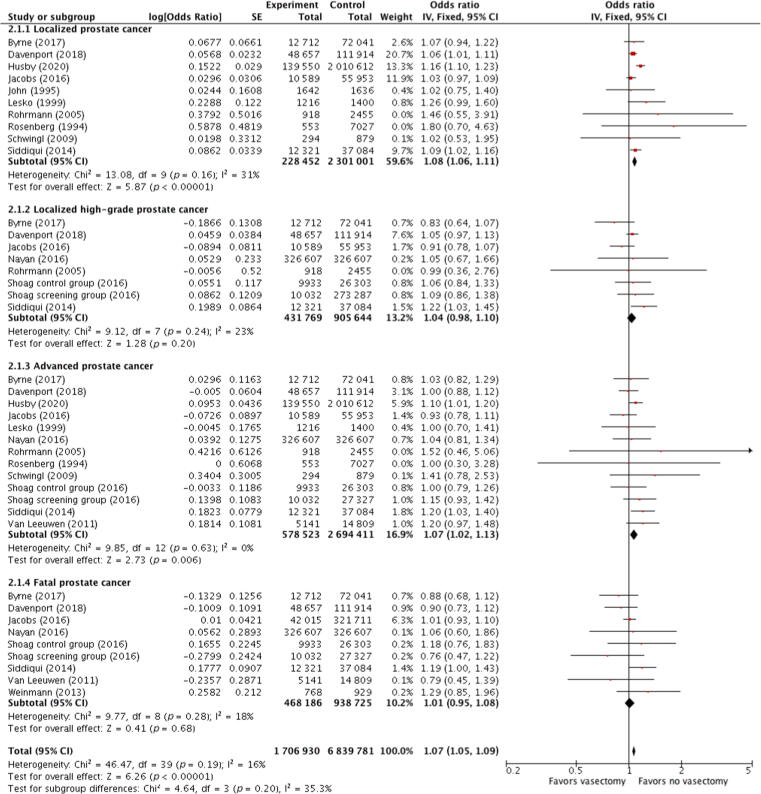
Forest plots showing the relation between vasectomy and prostate cancer by disease stage. Data were pooled separately by disease stage. As no significant heterogeneity (I^2^ < 50%) was found, a pooled estimate was calculated with the fixed-effect model (Mantel-Haenszel method). CI = confidence interval; df = degrees of freedom; IV = inverse variance; SE = standard error.

The association between vasectomy and localized, localized high-grade, advanced, and fatal PCa was investigated through the analyses of ten, eight, 13, and nine studies, respectively (Fig. 3). A significant association was found with localized PCa (OR 1.08; 95% CI, 1.06–1.11; *p* < 0.00001; I^2^ = 31%) and advanced PCa (OR 1.07; 95% CI, 1.02–1.13; *p* = 0.006; I^2^ = 0%). There was no significant association between vasectomy and localized high-grade PCa (OR 1.04; 95% CI, 0.98–1.10; *p* = 0.20; I^2^ = 23%) and PCa mortality (OR 1.01; 95% CI, 0.95–1.08; *p* = 0.68; I^2^ = 18%). The Cochrane’s Q and I^2^ tests did not show any heterogeneity in all pooled analyses.

### 3.6 Outcomes in PSA screening–adjusted studies

Seven studies reported an adjusted risk of PCa with PSA screening (five cohort and two case-control studies, *n* = 353 602). The pooled ORs remained significant for any (OR 1.06; 95% CI, 1.03–1.08; *p* < 0.0001; I^2^ = 10%) and localized (OR 1.06; 95% CI, 1.03–1.09; *p* = 0.0005; I^2^ = 0%) PCa. Conversely, there was a lack of association between vasectomy and localized high-grade (*p* = 0.19), advanced (*p* = 0.22), and fatal (*p* = 0.42) PCa (Table 2). The Cochrane’s Q and I^2^ tests did not show any heterogeneity in all pooled analyses. Indeed, the increase in any PCa risk decreased from 23% when all studies were considered to 9% when the analysis was limited to cohort studies, and to 6% for studies with a low RoB.

**Table 2:** Association between vasectomy and prostate cancer in studies adjusted with PSA screening

Analysis	Number of studies	Number of patients	RR (95% CI)	*p* value	I^2^ (%)
All prostate cancer	6	353 512	1.06 (1.03–1.08)	<0.0001	10
Localized prostate cancer	3	276 518	1.06 (1.03–1.09)	0.0005	0
Localized high-grade prostate cancer	5	350 113	1.05 (0.99–1.11)	0.19	35
Advanced prostate cancer	5	350 113	1.05 (0.97–1.13)	0.22	36
Fatal prostate cancer	6	351 810	1.03 (0.96–1.10)	0.42	33

CI = confidence interval; PSA = prostate-specific antigen; RR = relative risk.

### 3.7 Subgroup analyses

Restricting the analyses to studies published <10 yr ago, there was a significant association between vasectomy and any type of PCa (Table 3). The association remained significant when we restricted our analysis to studies with high quality (OR 1.06; 95% CI, 1.02–1.10; *p* = 0.02; I^2^ = 48%) or cohort studies (OR 1.09; 95% CI, 1.04–1.13; *p* = 0.0003; I^2^ = 64%). Among cohort studies, the association between vasectomy and PCa was significant only in studies with a mean/median follow-up duration of longer than 10 yr (OR 1.07; 95% CI, 1.04–1.11; *p* = 0.009; I^2^ = 56%). The Cochrane’s Q and I^2^ tests showed significant heterogeneity in all pooled analyses, except for high-quality studies.

**Table 3:** Subgroup analysis of the association between vasectomy and prostate cancer

Analysis	Number of studies	Number of patients	RR (95% CI)	*p* value	I^2^ (%)
Publication year					
1990–2000	10	186 275	1.20 (1.03–1.41)	0.001	68
2001–2010	10	261 997	1.22 (1–1.49)	<0.001	74
2011–2021	15	16 186 349	1.23 (1.04–1.44)	<0.001	98
Risk of bias					
Low	14	3 279 683	1.06 (1.02–1.10)	0.02	48
Intermediate/high	21	13 354 938	1.39 (1.07–1.80)	<0.001	97
Study design					
Cohort study	16	4 472 232	1.09 (1.04–1.13)	0.0003	64
Cross-sectional and case-control studies	19	12 162 389	1.36 (0.98–1.89)	<0.001	97
Follow-up (yr)					
<10	3	54 857	1.21 (0.79–1.85)	0.03	73
>10	12	3 543 890	1.07 (1.04–1.11)	0.009	56

CI = confidence interval; PSA = prostate-specific antigen; RR = relative risk.

### 3.8 Discussion

In this systematic review and meta-analysis, we found that vasectomy was significantly associated with a low risk of developing PCa. This association remained after restriction of our analyses to high-quality and cohort studies. However, the effect estimates of the association between vasectomy and PCa were increasingly closer to the null when analyzing studies with robust study design and study quality. Indeed, the increase in PCa risk fell from 23% when all studies were considered to 9% when the analysis was limited to cohort studies and to 6% for high-quality studies. It is questionable whether such low statistical significance may have a true clinical impact and whether it should influence vasectomy decision-making. It has been suggested that an individual cancer risk assessment could be considered before vasectomy, depending on other risk factors such as Afro-Caribbean origin or a family history of PCa [45]. Nevertheless, a statistically significant association is different from causation. Some preclinical studies have tried to explain the association [46], [47]. Possible explanations for the increased risk of PCa in vasectomized individuals include a decrease in prostatic secretory volume resulting in prolonged exposure to certain carcinogens, an increase in circulating androgens or in the binding capacity of androgen-binding proteins, development of antisemen antibodies that can affect immunological processes, and reduced levels of certain molecules in seminal plasma, such as IGF-1 and IGFBP3, known to be involved in prostate carcinogenesis. However, these molecular mechanisms underlying the link between vasectomy and PCa remain speculative. Therefore, we cannot argue with certainty that a causal association exists due to potential residual confounders.

Indeed, it has been suggested that men undergoing vasectomy likely have multiple factors that bias PCa detection, such as the intensity for follow-up PSA screening. Given the strong confounding effect of PSA screening, we assessed the risk of any PCa in studies adjusted for PSA screening. Vasectomy and PCa remained significantly associated but with an excess risk of only 6%, with no significant heterogeneity between the included studies. Compared with the last meta-analysis published by Xu et al [10], in a subgroup analysis including studies adjusted with PSA screening, we found no association between vasectomy and high-grade, advanced, or fatal localized PCa. Thus, our conclusions are more moderate and cautious than those of Xu et al [10]. At present, it is unknown whether residual confounding factors could be responsible for the modest excess of PCa incidence or whether this association should be considered definitive.

If assuming that PSA screening is a potential bias, it is expected to influence outcomes by disease stage. Indeed, PSA screening has been associated with increased detection of localized disease and decreased advanced PCa [48], [49]. Similar to two previous meta-analyses [10], [50], we found a positive association between vasectomy and advanced PCa. Nevertheless, as expected, when we restricted our analysis to studies adjusted for PSA screening, this association was no longer significant. Finally, PCa mortality was not influenced by vasectomy, which was consistent with previous reports [10], [50], [51], [52].

This review has several limitations that should be acknowledged. First, several confounding factors were not taken into account in the individual studies, making it impossible to establish definitively a causality between vasectomy and PCa. Second, substantial heterogeneity was observed across the included studies. Third, our results are based primarily on North American studies where vasectomy is more common (22% in Canada, 12% in USA, 11% in Oceania and Northern Europe, 3–5% in South America, and <1% in Africa [53]) and where PCa screening practices varied considerably over the study periods. Nevertheless, our study also has several strengths, including the number of patients included, the a priori definition of subgroup analyses, and the consideration of a detection bias by PSA screening for each PCa stage to refine the evaluation of the association.

### 3.9 Implications for practice and future research

The results of this study need to be interpreted with caution. Translating our results into clinical practice is likely to dissuade patients from undergoing vasectomy, whereas the absolute risk may be close to zero. Clear, fair, and understandable information should be provided about a possible association between vasectomy and PCa, without being able to determine whether there is any causality. To definitively address this question, future collaborative, well-designed, international studies are needed to prospectively assess this risk of PCa among vasectomized patients with particular attention to potential confounders such as well-established risk factors for developing PCa.

## 4 Conclusions

Our meta-analysis found a significant association between vasectomy and the risk of any, mainly localized, PCa. However, the effect estimates for the association between vasectomy and PCa were increasingly close to null when examining studies of robust design and quality. When we limited our analysis to studies adjusted for PSA screening, the association remained significant only for localized disease, but not for aggressive and/or advanced PCa. Future studies are needed to prospectively assess the possible causality between vasectomy and PCa, with attention to potential residual confounders that were not taken into account in large cohort studies.

  ***Author contributions*:** Michael Baboudjian had full access to all the data in the study and takes responsibility for the integrity of the data and the accuracy of the data analysis.

*Study concept and design*: Baboudjian, Ploussard.

*Acquisition of data*: Baboudjian, Rajwa, Ploussard.

*Analysis and interpretation of data*: Baboudjian, Rajwa, Ploussard.

*Drafting of the manuscript*: Baboudjian, Rajwa, Ploussard.

*Critical revision of the manuscript for important intellectual content*: Barret, Beauval, Brureau, Créhange, Dariane, Fiard, Fromont, Gauthé, Mathieu, Renard-Penna, Roubaud, Ruffion, Sargos, Rouprêt.

*Statistical analysis*: Baboudjian.

*Obtaining funding*: None.

*Administrative, technical, or material support*: None.

*Supervision*: None.

*Other*: None.

  ***Financial disclosures:*** Michael Baboudjian certifies that all conflicts of interest, including specific financial interests and relationships and affiliations relevant to the subject matter or materials discussed in the manuscript (eg, employment/affiliation, grants or funding, consultancies, honoraria, stock ownership or options, expert testimony, royalties, or patents filed, received, or pending), are the following: None.

  ***Funding/Support and role of the sponsor*:** None.
