# Supplementary material for: Vasectomy and Risk of Prostate Cancer: A Systematic Review and Meta-analysis
Source: Eur Urol Open Sci. 2022 May 19;41:35–44. doi: 10.1016/j.euros.2022.04.012 (PMC9130083; doi:10.1016/j.euros.2022.04.012)

**Supplementary Figure 2 – Funnel Plots for Publication Bias in the Studies Investigating Vasectomy and Prostate Cancer by Disease Stage**


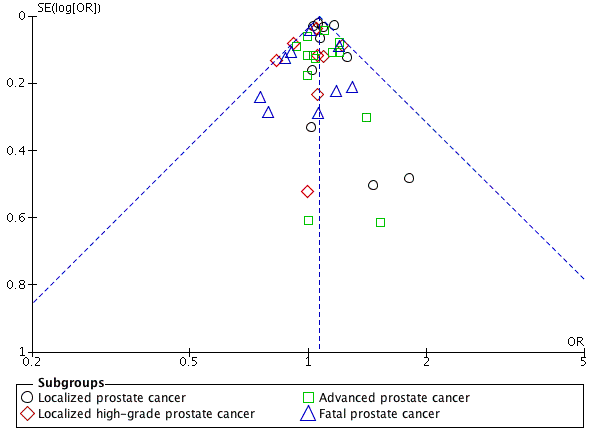

Supplement: Supplementary Figure 2 [file mmc2.docx]
