# Supplementary material for: Vasectomy and Risk of Prostate Cancer: A Systematic Review and Meta-analysis
Source: Eur Urol Open Sci. 2022 May 19;41:35–44. doi: 10.1016/j.euros.2022.04.012 (PMC9130083; doi:10.1016/j.euros.2022.04.012)
Supplement: Supplementary Table 1 [file mmc3.docx]

**Supplementary Table 1 – Newcastle-Ottawa Scale for Risk of Bias Assessment of Studies Included in**

**the Meta-Analysis**

| **Study** | **Selection** | | | | **Comparability** | **Outcome** | | | **Overall** |
| --- | --- | --- | --- | --- | --- | --- | --- | --- | --- |
|  | **Representativeness**  **of exposed cohort** | **Selection of nonexposed** | **Ascertainment of**  **exposure** | **Outcome not present**  **at start** |  | **Assessment of**  **outcome** | **Adequate follow-up**  **length** | **Adequacy of follow-up** |  |
| **Cohort and cross-sectional studies** | | | | | | | | | |
| **Byrne 2017** | 1 | 1 | 0 | 1 | 1 | 1 | 1 | 1 | 7 |
| **Davenport 2018** | 1 | 1 | 0 | 1 | 1 | 0 | 1 | 1 | 6 |
| **Eisenberg 2015** | 1 | 1 | 1 | 1 | 1 | 1 | 0 | 0 | 6 |
| **Giovannucci 1993** | 0 | 1 | 1 | 1 | 1 | 0 | 1 | 1 | 6 |
| **Goldacre 2005** | 1 | 1 | 1 | 1 | 1 | 1 | 1 | 1 | 8 |
| **Hiatt 1994** | 1 | 1 | 0 | 1 | 1 | 1 | 0 | 0 | 5 |
| **Husby 2020** | 1 | 1 | 0 | 1 | 1 | 1 | 1 | 1 | 7 |
| **Jacobs 2016** | 1 | 1 | 0 | 1 | 2 | 1 | 1 | 1 | 8 |
| **Lynge 2002** | 1 | 0 | 1 | 1 | 0 | 1 | 1 | 1 | 6 |
| **Nayan 2016** | 1 | 1 | 1 | 1 | 2 | 1 | 1 | 1 | 9 |
| **Rohrmann 2005** | 1 | 1 | 0 | 1 | 1 | 1 | 1 | 0 | 6 |
| **Seikkula 2020** | 1 | 0 | 1 | 1 | 0 | 1 | 1 | 1 | 6 |
| **Shoag 2016** | 1 | 1 | 0 | 1 | 2 | 1 | 1 | 1 | 8 |
| **Siddiqui 2014** | 1 | 1 | 0 | 1 | 2 | 1 | 1 | 1 | 8 |
| **Tangen 2016** | 1 | 1 | 0 | 1 | 1 | 1 | 1 | 1 | 7 |
| **Van Leeuwen 2011** | 1 | 1 | 0 | 1 | 2 | 1 | 1 | 1 | 8 |
| **Alqahtani 2015** | 1 | 1 | 0 | 1 | 0 | 1 | 0 | 0 | 4 |
| **De Antoni 1997** | 1 | 1 | 0 | 1 | 1 | 0 | 0 | 0 | 4 |
| **Study** | **Selection** | | | | **Comparability** | **Exposure** | | | **Overall** |
|  | **Case definition** | **Representativeness of**  **cases** | **Selection of controls** | **Control definition** |  | **Ascertainment of**  **exposure** | **Comparable**  **ascertainment method** | **Non-response rate** |  |
| **Case-control studies** | | | | | | | | | |
| **Cox 2002** | 1 | 1 | 1 | 1 | 1 | 1 | 1 | 1 | 8 |
| **Emard 2001** | 1 | 1 | 0 | 0 | 0 | 1 | 1 | 0 | 4 |
| **Hayes 1993** | 1 | 1 | 1 | 1 | 1 | 0 | 1 | 1 | 7 |
| **Hennis 2013** | 1 | 1 | 1 | 0 | 1 | 1 | 1 | 1 | 7 |
| **Holt 2008** | 1 | 1 | 1 | 0 | 2 | 0 | 1 | 1 | 7 |
| **John 1995** | 1 | 1 | 1 | 0 | 1 | 0 | 1 | 1 | 6 |
| **Lesko 1999** | 1 | 0 | 1 | 1 | 1 | 1 | 1 | 0 | 6 |
| **Lightfoot 2004** | 1 | 1 | 1 | 0 | 1 | 0 | 1 | 0 | 5 |
| **Mettlin 1990** | 0 | 1 | 0 | 0 | 1 | 0 | 1 | 0 | 3 |
| **Nair-Shalliker 2017** | 0 | 1 | 1 | 0 | 1 | 0 | 1 | 0 | 4 |
| **Patel 2005** | 1 | 1 | 1 | 0 | 1 | 0 | 1 | 1 | 6 |
| **Platz 1997** | 1 | 1 | 0 | 0 | 1 | 0 | 1 | 1 | 5 |
| **Romero 2012** | 1 | 1 | 0 | 1 | 0 | 1 | 0 | 0 | 4 |
| **Rosenberg 1994** | 1 | 1 | 0 | 1 | 1 | 0 | 1 | 1 | 6 |
| **Schwingl 2009** | 1 | 0 | 0 | 0 | 1 | 0 | 1 | 0 | 3 |
| **Stanford 1999** | 1 | 1 | 1 | 1 | 2 | 1 | 1 | 1 | 9 |
| **Sunny 2005** | 1 | 0 | 1 | 1 | 1 | 0 | 1 | 0 | 5 |
| **Weinmann 2013** | 0 | 1 | 0 | 1 | 1 | 0 | 0 | 0 | 3 |
